# *Treponema pallidum* Lipoprotein TP0435 Expressed in *Borrelia burgdorferi* Produces Multiple Surface/Periplasmic Isoforms and mediates Adherence

**DOI:** 10.1038/srep25593

**Published:** 2016-05-10

**Authors:** Kamfai Chan, Thayer Nasereddin, Laura Alter, Arturo Centurion-Lara, Lorenzo Giacani, Nikhat Parveen

**Affiliations:** 1 Department of Microbiology, Biochemistry and Molecular Genetics, Rutgers-New Jersey Medical School, Newark, NJ 07103, USA; 2 Department of Medicine, University of Washington, Seattle, WA 98104, USA.

## Abstract

The ability of *Treponema pallidum,* the syphilis spirochete to colonize various tissues requires the presence of surface-exposed adhesins that have been difficult to identify due to the inability to culture and genetically manipulate *T. pallidum*. Using a *Borrelia burgdorferi*-based heterologous system and gain-in-function approach, we show for the first time that a highly immunogenic lipoprotein TP0435 can be differentially processed into multiple isoforms with one variant stochastically displayed on the spirochete surface. TP0435 was previously believed to be exclusively located in *T. pallidum* periplasm. Furthermore, non-adherent *B. burgdorferi* strain expressing TP0435 acquires the ability to bind to a variety of host cells including placental cells and exhibits slow opsonophagocytosis *in vitro* similar to poor *ex vivo* phagocytosis of *T. pallidum* by host macrophages reported previously. This phenomenon of production of both surface and periplasmic immunogenic lipoprotein isoforms has possible implications in immune evasion of the obligate pathogen *T. pallidum* during infection.

Syphilis is a chronic, systemic, sexually transmitted disease that still affects millions of people worldwide. Syphilis is caused by the spirochete *Treponema pallidum* subspecies *pallidum* (*T. pallidum*), which was first isolated in 1912. Vertical transmission of this pathogen from mother to foetus is a leading cause of stillbirths in developing countries^1^,^2^. The molecular mechanisms of syphilis pathogenesis still remain poorly understood mostly due to inability to culture and genetically manipulate *T. pallidum*. Identification of *T. pallidum* lipoproteins as adhesins^3^,^4^ like those in other pathogenic spirochetes^5^,^6^,^7^,^8^ implies their surface localization^9^ and was instrumental in challenging the assertion that lipoproteins of *T. pallidum* reside exclusively in the periplasmic space and are not surface-exposed^3^,^10^,^11^,^12^,^13^,^14^,^15^,^16^,^17^,^18^,^19^,^20^. Weak labelling of the highly immunogenic *T. pallidum* lipoproteins by sera from patients and experimentally infected animals on the surface of these spirochetes^21^,^22^, and observation of low density of integral transmembrane protein complexes by freeze-fracture electron microscopy on outer membrane (OM) of *T. pallidum*^23^,^24^; however, support significant periplasmic location of the lipoproteins.

Heterologous expression systems using related bacterial species have been previously used to identify and functionally characterize virulence factors of various pathogens including adhesins, such as the ActA and Internalin proteins of *Listeria monocytogenes* and Invasin of *Yersinia pseudotuberculosis*^25^,^26^,^27^. Lyme disease-causing *Borrelia burgdorferi* and *T. pallidum* are structurally and physiologically related spirochetal pathogens that express different lipoproteins. Even though the stealth pathogen *T. pallidum* expresses fewer surface proteins, *B. burgdorferi* is a useful surrogate to express *T. pallidum* proteins, determine their sub cellular location, and investigate their maturation and function because; (a) lipoproteins are processed through similar biochemical pathways^28^; (b) 46% of *T. pallidum* open reading frames have orthologs in *B. burgdorferi* with potentially overlapping functions^29^; (c) both have characteristic spiral shape, possess flagella in the periplasmic space (endoflagella), and lack lipopolysaccharide (LPS); and (d) are extracellular pathogens that cause systemic diseases.

Some genomic and physiological differences exist between *T. pallidum* and *B. burgdorferi. B. burgdorferi* can be grown in complex medium *in vitro* and genetically manipulated^30^,^31^,^32^ and its genome (1.52 Mb) consists of a linear chromosome and numerous endogenous linear and circular plasmids^33^ encoding the majority of ~132 lipoproteins responsible for survival and colonization of tick vector and various hosts. In contrast, *T. pallidum* possesses a circular chromosome (1.13 Mb) and no plasmids^29^ and expresses only 22 putative lipoproteins with mostly still unconfirmed localization. Long-term *in vitro* cultivation of infectious *B. burgdorferi* strains results in the loss of its endogenous plasmids rendering the spirochetes non-adherent to host cell lines and non-infectious in the mouse model. We used two high passage, poorly adherent, non-infectious *B. burgdorferi* strains (B314 and B31HP), which have lost different endogenous plasmids^34^ to investigate the role of highly expressed and immunogenic TP0435. B314 has more severe loss of adhesins-encoding plasmids, such as Lp54 (Supplementary Fig. S1).

Early in our studies, we determined the structural similarities to predict potential roles of two major lipoproteins, TP0171 and TP0435 using Phyre 2 site and M4T^35^. TP0435 showed structural homology with the new lipoprotein E (NlpE) of *Escherichia coli,* a known adhesin. We selected TP0435 (also known as the 17 kD lipoprotein or Tpp17) for expression and to determine function of *T. pallidum* lipoprotein(s) using *B. burgdorferi* because of our interest in studying adherence mechanism of spirochetes. We show here that TP0435 is stochastically expressed on the surface of both *B. burgdorferi* and *T. pallidum* and this lipoprotein facilitates binding of the spirochetes to mammalian epithelial, glioma and placental cell lines.

## Results

### TP0435 is recognized by secondary syphilis patient serum on *B. burgdorferi* surface by Indirect Fluorescent Antibody (IFA) test

We cloned the *tp0435* gene along with its upstream 500 nucleotides containing putative promoter region in a *B. burgdorferi* shuttle vector, which also possesses a codon-optimized firefly luciferase gene^36^, and then used the construct to transform *B. burgdorferi* strains and denoted them as B314(pTP) and B31HP(pTP). B314 and B31HP strains transformed by vector alone designated as B314(V) and B31HP(V), respectively were also generated as control strains.

**Figure 1:**
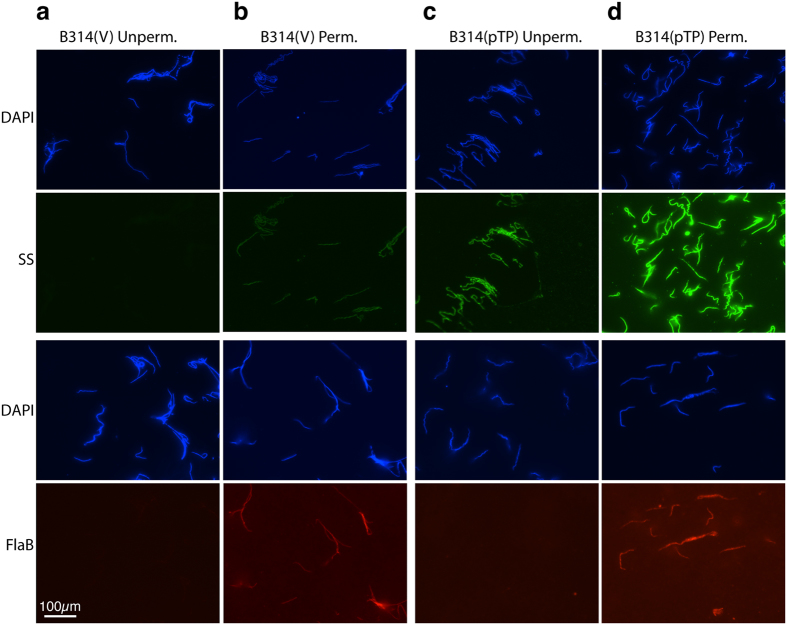
TP0435 is expressed on *B. burgdorferi* surface. **(a)** Absence of labelling of surface proteins of empty vector containing B314(V) control strain by secondary syphilis (SS) patient serum followed by treatment with anti-human Alexa fluor 488 conjugated secondary antibodies in IFA indicates that antibodies in this patient serum do not recognize *B. burgdorferi* surface proteins. All spirochetes in the respective fields were imaged after simultaneous staining with DAPI. Integrity of these bacteria during IFA was confirmed by lack of staining of periplasmic flagella with monoclonal antibodies against FlaB followed by reaction with anti-mouse TRITC conjugated antibodies. **(b**) Poor, albeit detectable, staining of B314(V) with SS patient serum on permeabilization indicates recognition of some periplasmic proteins of *B. burgdorferi* by this serum. (**c**) Punctate staining of majority of *B. burgdorferi* strain B314(pTP) with SS serum in IFA indicates the presence of TP0435 on spirochetes surface. Lack of periplasmic flagella staining again indicates that the integrity of outer membrane was maintained during IFA in this strain too. **(d**) Permeabilization of B314(pTP) results in more intense staining of TP0435 (Supplementary Table S1) indicating a significant presence of this protein also in the periplasmic region. Permeabilization also allowed intense staining of periplasmic flagella. Scale represents all panels in the figure.

IFA was conducted to assess whether antibodies in a syphilis patient serum detect TP0435 on intact *B. burgdorferi*. Surface labelling of both *B. burgdorferi* strains B314(pTP) and B31HP(pTP) and not control empty vector containing B314(V) and B31HP(V) strains (Fig. 1 and Supplementary Fig. S2) indicate that the patient serum recognizes TP0435 but not the surface proteins of *B. burgdorferi* strains used in this study. TP0435 expression, processing, and transport across the bacterial cytoplasmic membrane here validates *B. burgdorferi* as a useful surrogate system for *T. pallidum* lipoproteins. The absence of flagella staining without permeabilization indicates that the spirochetes remained intact during IFA (Fig. 1a, c bottom, and Supplementary Fig. S2a,c bottom Panels). Interestingly, permeabilization of B314(V) and B31HP(V) resulted in weak staining with secondary syphilis patient serum indicating some cross-reactivity with periplasmic protein(s). More intense staining of both TP0435 and periplasmic flagellin on permeabilization indicated that the majority of TP0435 is located in the periplasmic compartment of the spirochetes (Fig. 1d and Supplementary Fig. S2d). Indeed, on probing with syphilis patient serum, average fluorescence intensity did not change for B314(V) and B31HP(V) control strains after permeabilization, while it increased to 2–3 fold in B314(pTP) and B31HP(pTP) after permeabilization (Supplementary Table S1).

### TP0435 of *T. pallidum* produces multiple protein isoforms in *B. burgdorferi*

**Figure 2:**
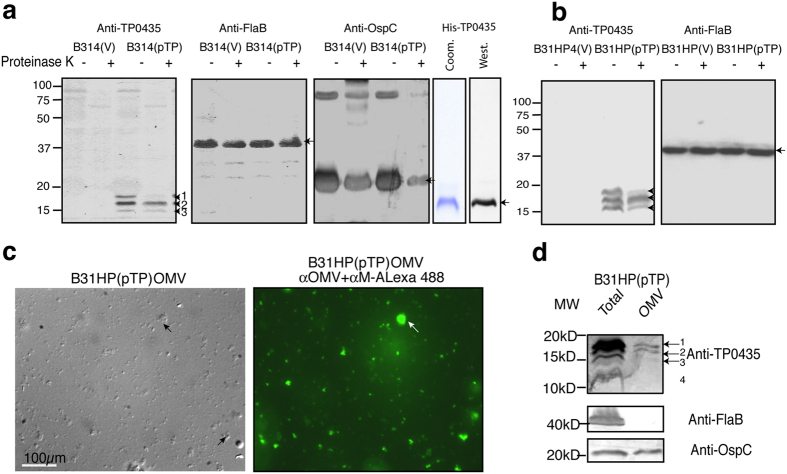
Expression of TP0435 lipoprotein of *T. pallidum* in *B. burgdorferi* produces multiple isoforms. **(a)** Analyses of total proteins of B314(V) and B314(pTP) with and without limited Proteinase K treatment were resolved by two SDS-PAGE gels with the same amount of respective samples loaded in duplicate in each gel. Western blotting of the gels using mouse anti-TP0435 serum showed the presence of multiple protein bands only when TP0435 is expressed. Limited Proteinase K treatment of intact *B. burgdorferi* cleaved at least the top TP0435 protein band number 1 (arrow) indicating that this isoform at minimum is surface exposed. Cleavage of protein band 3 likely also occurred to some extent. Negligible background staining indicates significant specificity of the mouse antibodies. Detection of intact FlaB (control for periplasmic proteins) and cleavage of the OspC (surface *Borrelia* lipoprotein) in the parallel blots confirms that only surface proteins are digested by limited Proteinase K treatment here. Arrows indicate FlaB and OspC protein monomers while higher oligomer of OspC is also observed. Clean preparation of recombinant TP0435 detected by Coomassie staining (Coom.) and Western blotting with anti-TP0435 serum (West.) with no bands equivalent to the isoforms seen in spirochetes preparation observed. **(b)** Results in ‘**a**’ were reproduced with B31HP(pTP) and B31HP(V) strains. **(c)** Clean Outer membrane Vesicles (OMV) preparation of B31HP(pTP) without contamination with spirochetal structures was obtained, as detected by Differential Interference Contrast (DIC, left) or by staining with anti-*B. burgdorferi* OMV mouse serum followed by anti-mouse (α-M) secondary antibodies conjugated to Alexa fluor 488 (right). Arrows indicate cluster of OMVs. (**d**) OspC of *B. burgdorferi* and three isoforms (bands 1, 2, and probably 3) of TP0435 remain associated with the OMV as detected by Western blotting. Absence of flagellin in OMV preparation confirms that the preparation is not contaminated with major periplasmic proteins. Lane marked as ‘Total’ indicates that the spirochetes total proteins were loaded in the lane.

Using a mouse antiserum that we raised against purified recombinant TP0435 for immunoblotting of whole *B. burgdorferi* B314(pTP) cells lysate, we detected multiple TP0435 isoforms ranging between 14–17 kD in size and are numbered starting with number 1 on the top, the largest protein band. The presence of protein isoforms is an unusual feature for bacterial exported proteins (Fig. 2a). Two protein gels were run and blotted in the same setup in duplicate and probed with three mouse antisera raised against spirochete proteins. Only weak cross reactivity of antibodies against TP0435 to control B314(V) strain indicates that the antiserum is specific for this protein. Limited Proteinase K treatment of intact live B314(V) and B314(pTP) strains indicated that OspC, a 21 kD surface lipoprotein of *B. burgdorferi* that is expressed at high levels in B314 strain, was digested but not the periplasmic flagellar protein FlaB, confirming that only surface proteins are susceptible to this treatment. Furthermore, susceptibility of at least the largest isoform (band 1), and potentially also band 3 to proteolysis indicates that TP0435 is exposed on the bacterial surface. Similar results were obtained when the B31HP(pTP) strain was treated in an identical manner (Fig. 2b) indicating that multiple isoforms are not an artefact of expression of TP0435 in a particular *B. burgdorferi* strain. Multiple bands equivalent to isoforms in spirochetes were not detected with purified recombinant TP0435 as detected by Coomassie stain or Western blotting (Fig. 2a, right panels). It is currently believed by several researchers that the outer membrane of *T. pallidum* is deficient in transmembrane proteins^23^ and lipoproteins are anchored towards the periplasmic leaflet of spirochete outer membrane. However, association of TP0435 with membrane fraction was confirmed by phase partitioning with non-ionic detergent, triton X-114^37^. Therefore, to determine whether different isoforms of TP0435 are also associated with the outer membrane of *B. burgdorferi* or may be anchored to its inner membrane or peptidoglycan layer in the periplasmic compartment, we purified outer membrane vesicles (OMV) from B31HP(pTP). Observation of purified OMV by Differential Interference Contrast (DIC, Fig. 2c left) or after staining with our anti-*B. burgdorferi* OMV antibodies followed by anti-mouse antibodies conjugated to Alexa fluor 488 (Fig. 2c right) indicated the complete absence of intact/ghost spirochetes in the preparation. SDS-PAGE resolution followed by Western blotting on loading total proteins from more than 10^8^ spirochetes proteins showed the presence of an additional 14 kD TP0435 band and at least top two isoforms association with the OMV with only a barely visible band 3 (Fig. 2d); however, we cannot rule out the possibility that due to low yield of highly purified OMV, the lack of lower band association with outer membrane is only due to sensitivity of detection. Detection of OspC and not periplasmic flagellin further confirmed purity of our OMV preparation.

### TP0435 is displayed on *B. burgdorferi* surface in a stochastic manner

**Figure 3:**
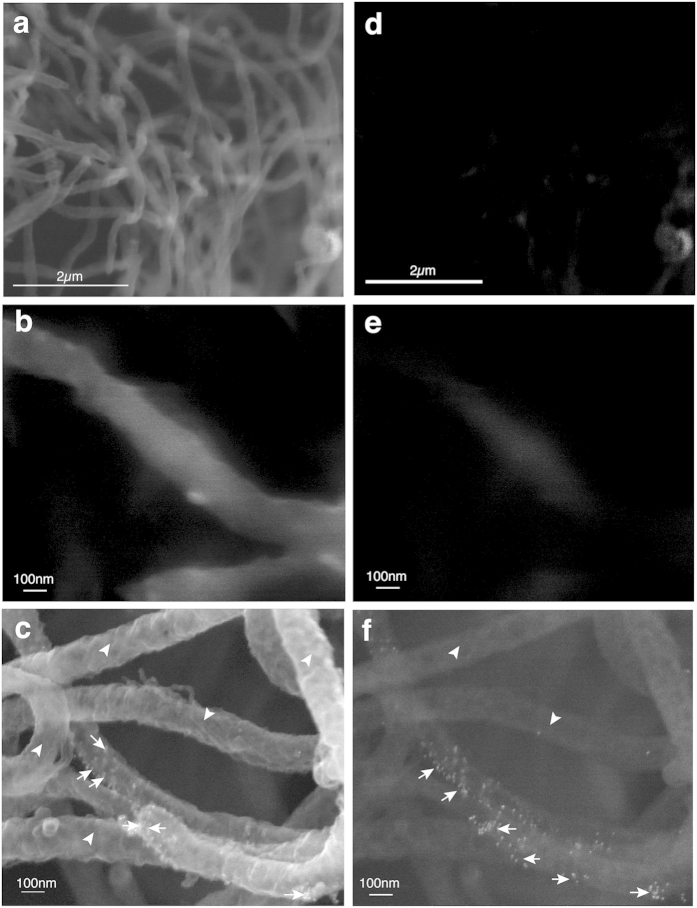
Immuno-SEM shows TP0435 is displayed on *B. burgdorferi* cells surface in a stochastic manner. (**a**,**b)** Absence of gold particles attached to B314(V) control spirochetes probed with secondary syphilis (SS) patient serum followed by anti-human gold conjugated antibodies (at 25x and 100x magnifications) confirms that this serum does not recognize surface proteins of *B. burgdorferi* strain B314. **(c)** Immuno-SEM imaging of B314(pTP) probed with SS serum at high magnification (100x) showed attached gold particles only on some of the *B. burgdorferi* cells (arrows). Arrowheads indicate the spirochetes without any gold particles attached. **(d**,**e)** Absence of attached gold particles by Back Scattered Imaging (BSI) of B314(V) in the field shown in the Fig. 3a,b, respectively confirms that the patient serum does not recognize surface proteins of B314 strain. **(f)** Stochastic expression of TP0435 on B314(pTP) surface in Fig. 3c is more clearly visible in BSI of the same field.

**Figure 4:**
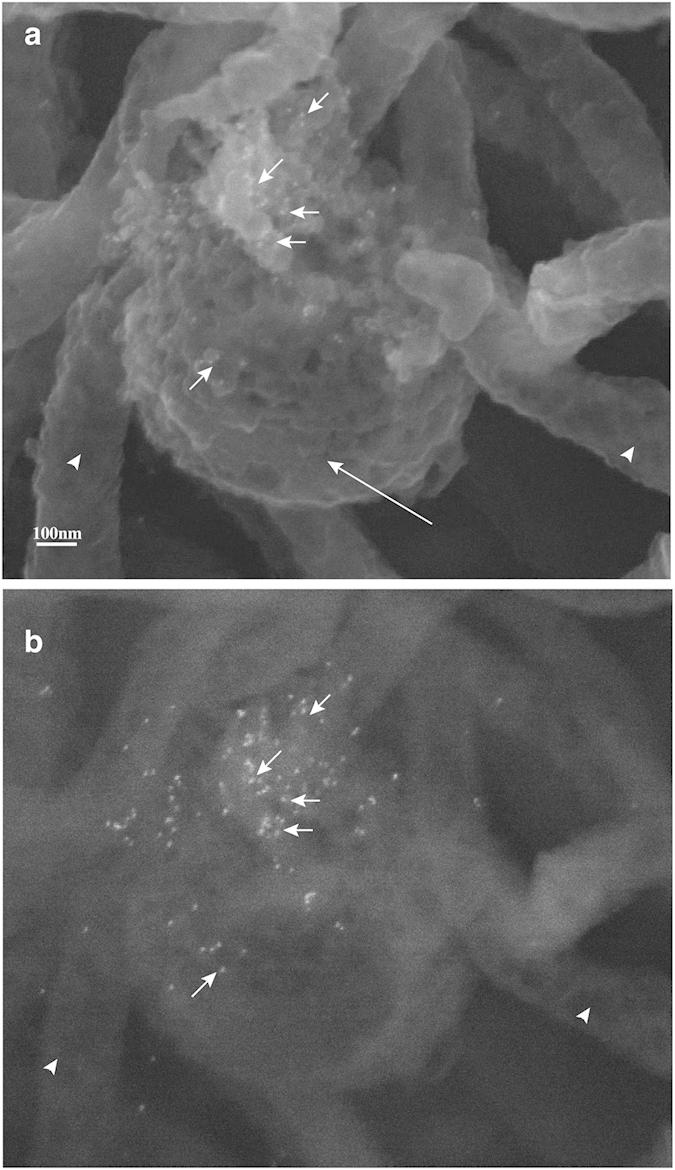
TP0435 is more prominently present in the inner, periplasmic face of the outer membrane (OM). **(a)** Inner concave face of accidently ripped OM of one *B. burgdorferi* shows more attached gold particles compared with those on the outer surface of another bacterium indicating that the most of TP0435 is present in periplasmic region during actively growing bacteria. Round structure indicated by a larger arrow indicates the convex outer surface of an OMV. **(b)** Attached gold particles in the field shown in **‘a’** are more clearly observed by the Back Scattered Imaging (BSI).

Since the pattern of expression of TP0435 is similar in both *B. burgdorferi* strains, further studies were primarily conducted using B314 strain. No gold labelling was observed on the *B. burgdorferi* control B314(V) strain probed with the same secondary syphilis patient serum by Immunogold-Scanning Electron Microscopy (Immuno-SEM) (Fig. 3a,b) even in back-scattered images (BSI) of the same field (Fig. 3d,e) confirming that antibodies in this serum do not recognize surface proteins of B314 strain. Interestingly, only a few *B. burgdorferi* cells in each field showed TP0435 scattered on their surface, as detected by the presence of bound gold particles (Fig. 3c). Gold particles are more clearly visible in the BSI of the same field of view of B314(pTP) (Fig. 3f). Stronger gold labelling was observed in the inner concave face of the outer membrane accessed through accidental tearing of the OM (Fig. 4a) than on the outer convex surface of the OMV observed in the same field. Again, stochastic labelling with the gold particles is more clearly observed by BSI (Fig. 4b).

To determine if antibodies raised in mice against recombinant TP0435, which lacked the first 25 amino acids of translated protein, can recognize the conformational epitopes on *B. burgdorferi* surface or periplasmic region, we conducted IFA (Supplementary Fig. S3). Anti-TP0435 serum showed high background fluorescence probably due to haemolysis of blood collected to recover serum, making detection of the surface protein on *B. burgdorferi* difficult. Punctate TP0434 could be detected by IFA following permeabilization.

### Stochastic distribution of TP0435 is also observed on *T. pallidum* surface

**Figure 5:**
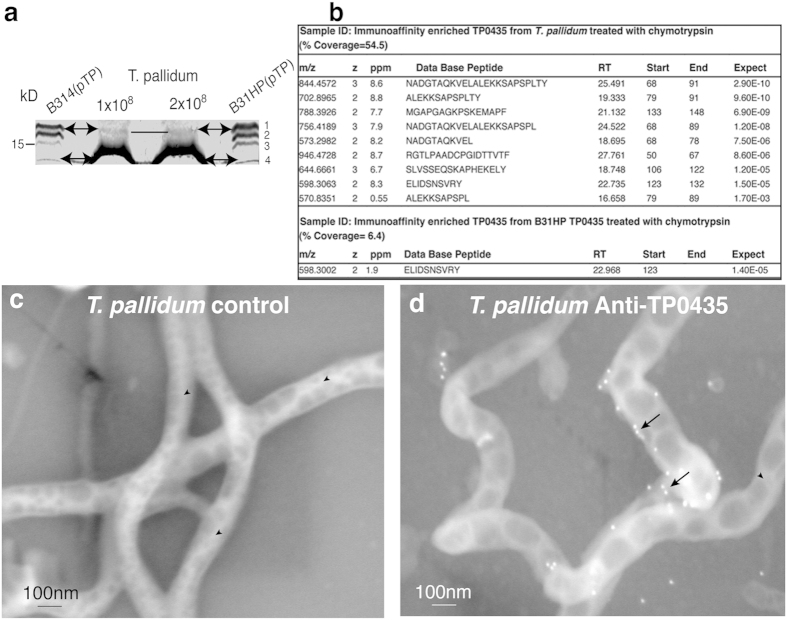
Random pattern of TP0435 surface expression is also observed in *T. pallidum* cells. **(a)** Protein bands likely equivalent to at least two isoforms (17 kD and 14 kD) detected in B314(pTP) and B31HP(pTP) strains are also discernible in *T. pallidum* by Western blotting. (**b**) Liquid Chromatography-tandem Mass-Spectrometric (MS/MS) analyses of TP0435 in *T. pallidum* Nichols strain and B31HP(pTP) after chymotrypsin digestion (Supplementary Fig. S5a). Table shows the derived peptide sequence, start and end of the peptide, parts per million (ppm), m/z representing mass (m) divided by charge number (z), retention time of the peptide in column (RT), and the best expected value the peptide (Expect). **(c)** Lack of attached gold particles on *T. pallidum* surface when probed with normal mouse serum followed by anti-mouse gold antibodies (arrowheads) shows that the prebled mouse serum or secondary antibodies do not non-specifically recognize *T. pallidum* proteins. **(d)** *T. pallidum* probed with anti-TP0435 mouse antibodies followed by secondary antibodies conjugated to gold particles indicate surface localization of TP0435 occur randomly in some *T. pallidum* cells (arrows).

Western blot analysis of total proteins of *T. pallidum* Nichols strain resolved by SDS-PAGE using mouse anti-TP0435 antibodies consistently showed the presence of at least two bands even though only the smallest protein is predominantly detected (Fig. 5a). TP0435 protein isoforms of different sizes in *B. burgdorferi* suggests differential processing of translated protein; however, the presence of isoforms in *T. pallidum* could not be ascertained. The level of ~14 kD TP0435 in *B. bu*rgdorferi is quite low indicating that some differences exist in the level of processed membrane proteins in two spirochetes possibly reflecting differences conferred by *in vitro* and *in vivo* growth conditions for *B. burgdorferi* and *T. pallidum*, respectively. In our experiments, the top ~17 kD protein band was detected in *T. pallidum* only when protein extract from more spirochetes (1–2 × 10^8^ per lane) was loaded in gel.

Antibodies present in secondary syphilis patient sera react with a variety of *T. pallidum* antigens. Therefore, to test whether TP0435 is indeed also present on the *T. pallidum* surface, immuno-SEM of *T. pallidum* was conducted using anti-TP0435 mouse serum. *T. pallidum* treated with mouse normal prebled serum did not show any attached gold particles (Fig. 5c). Surface labelling of only some *T. pallidum* spirochetes in each field of view was observed with anti-TP0435 mouse serum (Fig. 5d) and was similar to our immuno-SEM results with the B314(pTP) strain of *B. burgdorferi* when treated with secondary syphilis patient serum (Figs 3b and 4a). Labelling of TP0435 increased for *T. pallidum* harvests in which the outer membrane of spirochetes recovered from the rabbit testes was disrupted and accessibility of antibodies increased to the abundant TP0435 periplasmic isoforms (Supplementary Fig. S4a,b). Together these results suggest that *B. burgdorferi* and *T. pallidum* can both process newly translated TP0435 proteins to produce mature variants that are differentially localized either exclusively to the periplasmic space or to both the periplasm and the surface of the spirochetes. Surface display of TP0435 in each spirochete appears random (Figs 3c,4a and 5d) indicating that TP0435 post-translational processing in each bacterial cell present in a population occurs independently and stochastically.

### Post-translational processing likely produces multiple TP0435 isoforms

We were unable to sequence TP0435 immuno-enriched from *B. burgdorferi* strain and resolved by SDS-PAGE by Edman degradation suggesting that the NH_2_-terminal end of TP0435 is blocked. Suggested localization of the protein isoforms in the outer membrane versus inner membrane by LipoP1.0 program falls between 30–70%. Liquid Chromatography and Tandem Mass Spectrometric (LC-MS/MS) analyses of TP0435 after enrichment from *T. pallidum* and B31HP by immunoaffinity chromatography detected different peptides of TP0435 after chymotrypsin digestion (Fig. 5b and Supplementary Fig. S5a); however, did not recognize the N-terminal modified/unmodified peptides. Identification of peptides, modified and unmodified by MS analyses depends on known sequence and mass. Differential processing at different Cysteine residues followed by lipidation could be responsible for multiple TP0435 isoforms production (Supplementary Fig. S5a,b). Interestingly, unlike *B. burgdorferi*, several *T. pallidum* predicted lipoproteins show multiple cysteines presence within the first 50 amino acids of translated Open Reading Frames (Supplementary Table S2). Since the nature of the lipid moiety attached to the NH_2_-terminal cysteine in TP0435 and its mass is not known, we were unable to determine the sequence of NH_2_-terminal lipidated peptides in the isoforms by MS analyses of either *T. pallidum* or B31HP(pTP) despite trying four different MS core facilities of well-known universities in the USA. Furthermore, it is difficult to obtain milligrams of purified proteins from spirochetes required for identification of lipidated peptides.

### TP0435 is an adhesin that facilitates binding to the mammalian cells

The next logical step was to determine the role of the surface localized TP0435 in spirochete pathogenesis using a tissue culture system. To conduct these studies, the structural homology of TP0435 protein with *E. coli* NlpE (new lipoprotein E) protein was used as foundation because the crystal structure of NlpE and its function were already determined^38^. Using BLAST, NlpE shows 35% of sequence identity and 48% similarity with TP0435. Additionally, Phyre 2 program predicted TP0435 structural similarity with NlpE at 99.97% confidence. The predicted structure closely resembles the monomer TP0435 crystal structure determined very recently^39^ encouraging us to assess if TP0435 has a role in adherence.

**Figure 6:**
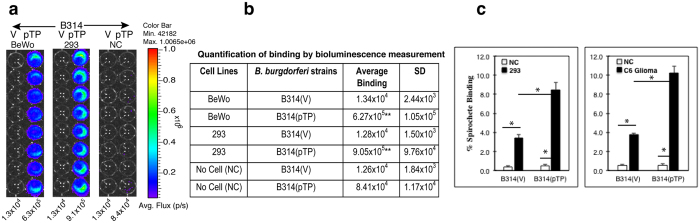
TP0435 is an adhesin that facilitates binding to epithelial, Glial and placental cells. **(a)** B314(pTP) strain is adherent to BeWo and HEK 293 cells with 50- and 70-fold increase in binding as compared to the control B314(V) strain to these cells as detected by measurement of light emitted by the attached luminescent spirochetes. NC indicates ‘No Cell’ control. **(b)** Adherence levels depicted in **‘a’** are quantified. Attachment of B314(pTP) is significantly higher than B314(V) binding to the respective cell lines as determined by student T test (**p < 0.001). Binding of B314(V) to both cell lines is not significantly different from B314(pTP) strain binding to NC control. **(c)** Binding of ^35^S-labeled B314(pTP) strain to HEK 293 and rat C6 Glioma cells increased in statistically significant manner as compared to B314(V) binding (*P < 0.001 by student T test). Binding of B314(V) to both cells was also significantly higher (*P < 0.001) as compared to binding of this strain to NC control.

To determine whether TP0435 is also an adhesin, we examined the ability of B314(pTP) strain to bind to embryonic human kidney epithelial 293 and placental BeWo cell lines. Bioluminescence measurement of *B. burgdorferi* B314 expressing TP0435 showed a statistically significant, 50–70-fold increase in binding to these two cell lines as compared to the B314(V) control strain (Fig. 6a,b), confirming TP0435 involvement in attachment to human cells. Because firefly luciferase activity is also sensitive to the pH of the medium and available ATP levels^40^, we also used a complementary technique to determine binding using ^35^S methionine-cysteine labelled *B. burgdorferi* strains (Fig. 6c). Again, B314(pTP) showed a statistically significant higher level of binding to both 293 and C6 Glioma cell lines as compared to the control B314(V) strain. Unfortunately, BeWo cells did not bind to the NUNC break apart plates used for these assays affecting integrity of the monolayers during washing and thus, binding of labelled *B. burgdorferi* (data not shown). However, our gain-of-function results determined by two complementary techniques with 293 cells included in both assays indicate that TP0435 present on the spirochete surface indeed functions as an adhesin (Fig. 6a,c).

### Slow opsonophagocytosis of *B. burgdorferi* strains expressing TP0435 similar to that observed in *T. pallidum*

**Figure 7:**
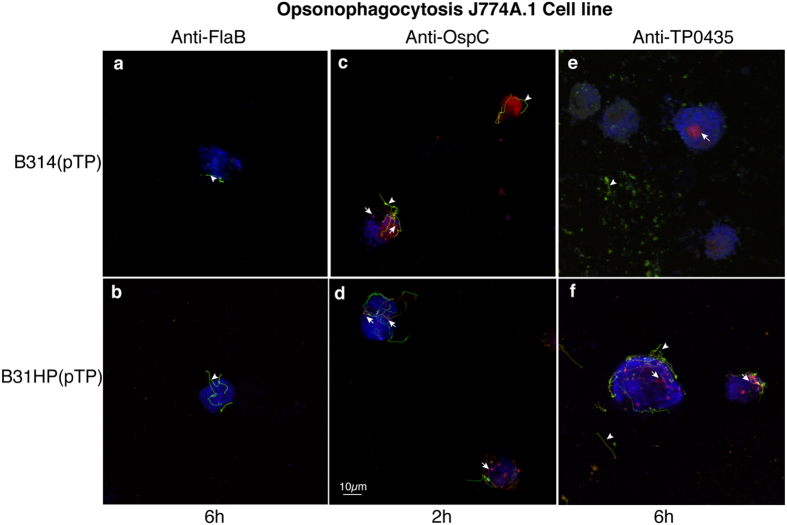
Anti-TP0435 mouse antibodies facilitate only low opsonophagocytosis of B314(pTP) and B31HP(pTP) strains. **(a**,**b)** J774A.1 mouse macrophage cell line failed to phagocytise B31HP(pTP) and B314(pTP) preincubated with antibodies against periplasmic protein FlaB even after 6 h of co-incubation. Extracellular spirochetes (green/yellow marked by arrowhead) were observed on staining of *B. burgdorferi* with anti-*B. burgdorferi* OMV antibodies followed by Alexa fluor 488 conjugated anti-mouse antibodies before permeabilization, while to detect intracellular spirochetes, after permeabilization counterstaining was done with anti-OMV antibodies followed by anti-mouse antibodies conjugated to TRITC. **(c**,**d)** Preincubation of B314(pTP) and B31HP(pTP) with antibodies against *B. burgdorferi* surface protein OspC showed phagocytosis by J774A.1 cells within 2 h of co-incubation shown as red intracellular degrading spirochetes. **(e**,**f)** B31HP(pTP) and B314(pTP) preincubated with anti-TP0435 antibodies bind to J774A.1 mouse macrophage cell line. After 6 h of co-incubation, both bound or unbound extracellular *B. burgdorferi* (green marked by arrowhead) and intracellular spirochetes (red marked by arrow) are observed indicating slow but detectable opsonophagocytosis. Scale represents all panels in the figure.

*B. burgdorferi* strains surrogate system also helped us detect phagocytosis-mediated killing by mouse macrophage cell line J774A.1. After incubation of B314(V), B314(pTP), B31HP(V) and B31HP(pTP) with the specific mouse antibodies, spirochetes were added to the macrophage cells, incubated at 37 ^o^C, fixed, stained and examined microscopically (Fig. 7). Extracellular spirochetes are green and were stained by respective antibodies prior to permeabilization while red fluorescence was obtained due to post-permeabilization staining and indicates phagocytosed spirochetes. In our negative control, J774A.1 macrophage cell line failed to phagocytise B314(pTP) and B31HP(pTP) strains preincubated with antibodies against periplasmic FlaB protein even after 6 h of co-incubation of bacteria with J774A.1 cells (Fig. 7a,b). Phagocytosis occurred within 2 h of co-incubation of macrophage cell line and B314(pTP) and B31HP(pTP) spirochetes preincubated with antibodies against *B. burgdorferi* surface protein OspC (Fig. 7c,d) indicating normal phagocytosis rate when antibodies against surface *B. burgdorferi* protein are used for opsonization. Bright red phagocytised and often degraded spirochetes were detected by staining after permeabilization of cells after 6 h co-incubation of J774A.1 cells with B314(pTP) or B31HP(pTP), which were opsonized with anti-TP0435 antibodies (Fig. 7e,f). Both of these spirochete strains also lost bioluminescence significantly when incubated with J774A.1 for 4 h indicating killing after phagocytosis (data not shown). These results suggest that *B. burgdorferi* cells expressing TP0435 were phagocytised and ultimately degraded even though opsonophagocytosis occurred rather slowly. Control *B. burgdorferi* strains with empty vector, B314(V) and B31HP(V), preincubated with anti-TP0435 serum remained extracellular (green) even after 6 h of co-incubation with macrophage cell line (Supplementary Fig. S6). Our results with non-infectious *B. burgdorferi* here also agree with previous findings with *T. pallidum* where opsonophagocytosis occurs at a slow rate compared to that observed for other pathogens likely due to limited surface exposure of these proteins^41^.

## Discussion

Receptor-mediated adherence to host cells plays a critical role in the life of extracellular pathogens that can persist in the host for long durations despite stimulation of a strong adaptive immune response. *T. pallidum* long-term survival in the host, which can lead to the serious clinical manifestations of tertiary syphilis is suggested to be facilitated by the uncommon paucity of proteins on the surface of this spirochete^3^,^10^,^11^,^12^,^13^,^14^,^15^,^16^,^17^,^18^,^19^,^20^. However, this scenario does not address how *T. pallidum* colonizes various human tissues during syphilis.

Our studies support that *B. burgdorferi* is a suitable heterologous system to functionally characterize *T. pallidum* proteins and possible virulence factors. Detection of multiple TP0435 isoforms in *B. burgdorferi* was unexpected and may allow escape of spirochetes from immune response. Different protein isoforms originating from the same precursor by differential post-translational processing and modification are not common in bacteria. Indeed, Lpp of a laboratory strain of commensal *E. coli* is the only other known bacterial lipoprotein that possesses two isoforms^42^,^43^, one located in the periplasmic space with a C-terminal lysine anchored to the peptidoglycan layer (bound form), while the second form is integrally associated with the outer membrane and is surface-exposed, even though its transmembrane domain(s) is yet to be identified^42^. NH_2_-terminal of both forms was suggested to be acylated, indicating that both versions are lipoproteins. Interestingly, C-terminal lysine residue is also present in TP0435 (Supplementary Fig. S5a); however, the experimental evidence for the exact anchor for periplasmic TP0435 is not yet available.

OMV preparations of *B. burgdorferi* expressing TP0435 show the presence of multiple isoforms associated with outer membrane. Top surface band (~17 kD, band number 1) appears to represent less than one third of the total TP0435 present in *B. burgdorferi* (Fig. 2a,b). This observation strongly suggests that a majority of TP0435 is periplasmic with only a smaller fraction being surface exposed. Low abundance of surface TP0435 protein in *T. pallidum,* which could be equivalent to the isoform 1 of TP0435 present in *B. burgdorferi,* was detected only after overloading the gel (Fig. 5a). A caveat of comparing the levels of each isoform present in the spirochetes by Western blotting is that antibodies against a specific epitope present only in a particular isoform may be missing and thus, could diminish detection of this isoform. For example, limited Proteinase K treatment suggests that band 3 of TP0435 may also be displayed on the surface to some extent but its quantity, as detected by Western analysis, appears to be low (Fig. 2). Even then, our findings confer with previous suggestion by other researchers that a significant amount of *T. pallidum* proteins are present in periplasmic region of the spirochetes recovered from rabbit testes^11^,^23^. A possible explanation is that a majority of the free spirochetes recovered from rabbit testes are those unable to bind to the host tissues and possess only a small amount of this lipoprotein displayed on the outer membrane surface^44^. Supporting this premise, we observed that TP0435 is stochastically expressed on the surface of only some *T. pallidum* cells (Fig. 5c). Based upon three adjacent or overlapping lipoboxes in TP0435, we postulate that differential cleavage of this surface immunogenic exported protein followed by lipidation could be a critical and unique mechanism for *T. pallidum* to produce lipoprotein isoforms. Only a limited amount of the outer membrane associated TP0435 then results in exposure on bacterial surface (Figs 2,3,4 and 5). We suggest that a limited surface exposure versus complete periplasmic presence of TP0435 in different *T. pallidum* cells during infection may ultimately lead to alternative pathogen fates.

Adherence through NlpE protein activates the CpxR/CpxA two component system and has been implicated in suppressing the extracytoplasmic toxicities triggered by mutant or misfolded outer membrane protein(s) in *E. coli*^45^. This suppression is achieved by up regulation of the production of the periplasmic protease, DegP, which degrades defective proteins^46^. Protein turnover in the periplasmic space could also be important in *T. pallidum*, considering that most of its lipoproteins are suggested to be present in this subsurface compartment. TP0435 is likely a bifunctional protein, similar to that observed for many other spirochete surface proteins^47^,^48^,^49^, with different roles played by surface and periplasmic versions of TP0435. Previously, two roles proposed for periplasmic TP0435 were: (i) redox sensor; or, (ii) communication of its redox/oligomerization state to downstream effectors^39^. We predict that immunogenic TP0435 location predominantly in the periplasmic space of the majority of disseminating *T. pallidum* cells particularly later in infection allows long-term sustenance of this obligate pathogen in the host, which is a necessary condition to develop late syphilis manifestations. Based upon our opsonophagocytosis results with *B. burgdorferi* and (Fig. 7) and slow phagocytosis of only a subpopulation of *T. pallidum* previously observed^41^,^50^, we postulate that the spirochetes that express TP0435 on their surface during the early stage of infection mediate adherence to various host cells. These *T. pallidum* cells are still protected in the blood stream since adaptive immune response is not yet established against its immunogenic proteins including TP0435. After development of a specific humoral response, *T. pallidum* cells that display TP0435 on their surface are negatively selected in the bloodstream, and only bacteria that lack TP0435 on their surface survive during dissemination. Thus, by the time an adaptive immune response against TP0435 is established, it is no longer needed on the surface of free *T. pallidum* while those still expressing TP0435 on the surface remain attached to the target cells, facilitating colonization of the specific tissues probably in the immunoprivileged sites.

## Methods

### Ethics Statement

New Zealand white rabbits were used for *T. pallidum* strain propagation. Animal care was provided in accordance with the Guide for the Care and Use of Laboratory Animals and procedures were conducted under protocols approved by the University of Washington Institutional Animal Care and Use Committee (IACUC) under protocol number 4243–01. Polyclonal antibodies against recombinant TP0435 were produced in BALB/c mice using the previously described protocol^1^. All mouse experiments were performed in accordance with all provisions of the Animal Welfare Act, the Guide for the Care and Use of Laboratory Animals, and the PHS Policy on Humane Care and Use of Laboratory Animals. These experiments were conducted under the protocol number 14011D0617 approved by the Rutgers Biomedical and Health Sciences IACUC. Coded, secondary syphilis patient serum was provided for this work by Dr. Sheila Lukehart.

### Cultivation of *B. burgdorferi*

High passage *B. burgdorferi* strains B314 and B31HP^34^ were grown in BSKII medium containing 6% rabbit serum at 33 ^o^C.

### Isolation and purification of Outer membrane Vesicles (OMV) of B31HP(pTP)

OMV were separated from cytoplasmic cylinder using citrate buffer and purified by sucrose gradient centrifugation as described previously^48^,^51^.

### Production of antibodies in mice against recombinant TP0435

Antibodies were raised against polyhistidine tagged recombinant purified TP0435 in BALB/c mice using our previously described protocol^48^. Detailed method used, with minor modifications in the previously described protocol, is provided in the Supplementary information.

### *T. pallidum* strain propagation

*T. pallidum* Nichols strain^52^, originally provided by James N. Miller, University of California, Los Angeles, CA, was propagated in New Zealand white rabbits by means of intratesticular (IT) inoculation as previously reported^53^ and described in the supplementary information.

### Mammalian cells culture

C6 (rat) glioma and human embryonic kidney 293 cell lines in our collection (originally obtained from Dr. John Leong’s laboratory at Tufts University School of Medicine) were grown at 37 °C and 5% CO_2_ atmosphere as previously described^54^. BeWo cells (obtained from American Type Culture Collection) were cultured in F-12K medium supplemented with 10% FBS and Penicillin/Streptomycin (P/S) mixture. Mouse macrophage J774A.1 cell line was grown in Dulbecco’s Modified Eagle’s Medium (DMEM) supplemented with 10% FBS and P/S mixture, except antibiotic mixture was eliminated during the J774A.1 cells co-incubation with *B. burgdorferi* strains for phagocytosis experiment. DMEM, FBS and BSA were obtained from commercial sources.

### Immunoaffinity enrichment of TP0435

After washing *B. burgdorferi* B31HP(pTP) culture three times with the suspension buffer (50 mM Tris-HCl, pH7.4, 150 mM NaCl, 5 mM EDTA), lysis was carried out with 1.7% TritonX100 containing suspension buffer i.e., lysis buffer, in the presence of protease inhibitor cocktail (Sigma P8849). After removing lysed bacterial pellet by centrifugation, proteins in the supernatant were precipitated with three volumes of cold acetone and after drying in air, pellet was resuspended in solubilization buffer, i.e., suspension buffer containing 0.2% Triton-X100 and protease inhibitor cocktail. Immunoaffinity enrichment of TP0435 from *B. burgdorferi* was conducted using a two-step process. In the first step, antibodies against *B. burgdorferi* cross-linked with protein G were used to remove majority of Borrelia proteins and supernatant containing significantly reduced level of *B. burgdorferi* proteins recovered after centrifugation. Protein G beads crosslinked to TP0435 mouse antiserum were then used to further enrich TP0435 protein from this supernatant. After washing, the beads boiled in protein loading dye were resolved by 12.5% SDS-PAGE and stained with Sypro Ruby (*T. pallidum*) or silver stain (*B. burgdorferi*). The first enrichment step was eliminated for immunoprecipitation and enrichment of TP0435 from *T. pallidum*. Gel segment containing enriched TP0435 from *B. burgdorferi* (4–5 mm) was sent to Tufts University Core facility to determine N-terminal sequence by Edman degradation method.

### Protein Identification Using Reversed-phase Liquid Chromatography Electrospray Tandem Mass Spectrometry (LC-MS/MS)

A 4–5 mm segment of gel region containing TP0435 was excised from the gel and subjected to in-gel digestion with high fidelity chymotrypsin. The peptides formed from the digested samples were analyzed by on-line LC-MS/MS technique. The LC separation was performed using a NanoAcquity UPLC system (Waters) on an Easy-Spray PepMap® column (75 um × 15 cm, Thermo Scientific) with a linear gradient from 2–30% B (0.1% formic acid in acetonitrile) followed by washing at 50% B at a flow rate of 600 nl/min. The MS/MS analysis was performed using a LTQ Orbitrap Velos mass spectrometer (Thermo Scientific). After a survey scan, 6 most intense precursor ions were selected for subsequent fragmentation using HCD with normalized collision energy of 30. Both precursor and fragment ions were analyzed in the FT mode in the Orbitrap at mass resolution of 30000 and 7500, respectively. The analytical peak lists were generated from the raw data using an, in-house software, PAVA^55^. The MS/MS data were searched against the UniProt database using a University of California at San Francisco search engine, Protein Prospector, available at http://prospector.ucsf.edu/prospector/mshome.htm.

### IFA and Immuno-SEM of *B. burgdorferi* and *T. pallidum*

IFA of *B. burgdorferi* was conducted using previously reported protocol that is briefly described in the supplementary information. After centrifugation of *B. burgdorferi* grown to mid logarithmic phase at low speed (4,000 × g), the medium was discarded and culture fixed with 16% paraformaldehyde solution (Electron Microscopy Sciences, Hatfield, PA) for 20 more minutes with light vortex every five minutes. Bacteria were washed twice with sodium phosphate buffer (0.2 M concentration, pH7.2) containing 0.2% BSA (PBS/BSA). After incubation with the secondary syphilis patient serum overnight at 30 ^o^C, culture was washed three times with PBS/BSA. Bacteria were incubated at 30 ^o^C with goat anti-human IgG antibody conjugated with 10 nm gold particles used at 1:3 dilutions (Abcam, MA) for 2 h. After three washings with PBS/BSA, spirochetes were fixed in 2.5% glutaraldehyde for 30 minutes at room temperature and then dehydrated with a series of ethanol. After dehydration, culture pellets were dried in liquid CO_2_ (Tousimis Critical Point Dryer, Autosamdri-815, MD). Dried bacteria were then mounted onto aluminium SEM stubs with conductive carbon tape and then coated with 1.5 nm of carbon in a Denton Vacuum Evaporator 502B (Moorestown, NJ), and the specimens were examined and pictures taken by Gregory Hendricks at the University of Massachusetts Medical School, Core EM Facility using an FEI Quanta 200 FEG MK II scanning electron microscope.

Fresh *T. pallidum* cells were processed in similar manner except that the specific mouse polyclonal antiserum raised against TP0435 was used followed by a goat anti-mouse secondary antibody conjugated with 10 nm gold particles (Sigma-Aldrich, St. Louis, MO). Bacteria mounted on carbon SEM stubs (Electron Microscopy Sciences, Hatfield, PA) were coated with carbon in a Denton Vacuum Evaporator DV-502 (Moorestown, NJ) and images taken by Geoffrey Perumal at the Analytical Imaging Facility of Albert Einstein College of Medicine using a Zeiss Supra 40 Field Emission Scanning Electron Microscope (Carl Zeiss Microscopy, LLC, North America), with a backscatter detector using an accelerating voltage of 10 KV.

### Binding assays

For determination of binding by bioluminescence detection, *B. burgdorferi* cells were counted and concentration adjusted to ~3.7 × 10^7^ cells/ml. Fifty microliter of culture suspension was added per well of a 96-well BD Falcon plate with black walls containing mammalian cell line monolayers. After centrifugation at 233 × g for 5 minutes, the plate was incubated at 37 °C with 5% CO_2_ for 1 hour to allow binding of bacterial cells. After three washes with 150 μl PBS/BSA (five minutes) without shaking, 100 μl of BSKII-RS containing 0.15 mg/ml D-luciferin was added to each well. Bioluminescence was measured using an IVIS-50 instrument (Perkin-Elmer, MA). Binding assays with radiolabel *B. burgdorferi* were carried out using previously described protocol^56^.

### Phagocytosis of *B. burgdorferi* expressing TP0435

B314(pTP) and B31HP(pTP) and controls, B314(V) and B31HP(V), were incubated for 30 minutes with anti-TP0435 polyclonal mouse antibodies diluted 1:100 in J774A.1 medium. As positive and negative controls, B314(pTP) and B31HP(pTP) were preincubated with antibodies against surface and periplasmic *B. burgdorferi* proteins OspC and FlaB, respectively. After incubation of mouse macrophage J774A.1 cell line monolayers at 50% confluence with opsonised bacteria in medium without antibiotics for 2 h, 3 h, and 6 h at 37 ^o^C in 5% CO_2_ incubator (also 4 h for bioluminescence measurement only), co-incubated cells were fixed for 1 h using 3% paraformaldehyde in PBS. After fixation and blocking with PBS containing 5% BSA and 5% heat inactivated goat serum, samples were incubated with antibodies raised against crude OMV preparation of *B. burgdorferi* followed by secondary antibodies conjugated to Alexa fluor 488 to label extracellular spirochetes. We had already raised antibodies against crude OMV preparation from infectious *B. burgdorferi* strain in BALB/c mice using the protocol described previously^48^. J774A.1 cells were then stained with wheat agglutinin lectin conjugated with Alexa fluor 647. Cells were then permeabilized with cold methanol and *B. burgdorferi* counterstained with the anti-OMV primary antiserum followed by a secondary antiserum conjugated with TRITC to stain phagocytised bacteria.

## Additional Information

**How to cite this article**: Chan, K. *et al.* *Treponema pallidum* Lipoprotein TP0435 Expressed in *Borrelia burgdorferi* Produces Multiple Surface/Periplasmic Isoforms and mediates Adherence. *Sci. Rep.* **6**, 25593; doi: 10.1038/srep25593 (2016).

## Supplementary Material

Supplementary Information
