# Supplementary material for: Treponema pallidum Lipoprotein TP0435 Expressed in Borrelia burgdorferi Produces Multiple Surface/Periplasmic Isoforms and mediates Adherence
Source: Sci Rep. 2016 May 10;6:25593. doi: 10.1038/srep25593 (PMC4861935; doi:10.1038/srep25593)
Supplement: Supplementary Information [file srep25593-s1.pdf]

***Treponema pallidum* Lipoprotein TP0435 Expressed in *Borrelia burgdorferi* Produces Multiple Surface/Periplasmic Isoforms and mediates Adherence**

Short title: TP0435 Post-translational Isoforms and Adherence

Authors: Kamfai Chan[1‡], Thayer Nasereddin[1‡], Laura Alter[1], Arturo Centurion-Lara[2†], Lorenzo Giacani[2] and Nikhat Parveen[1]*

[1]Department of Microbiology, Biochemistry and Molecular Genetics, Rutgers-New Jersey Medical School, Newark, NJ 07103, USA.
[2]Department of Medicine, University of Washington, Seattle, WA 98104.

‡These authors contributed equally to this work

Supplementary Information

Methods

Ethics Statement. New Zealand white rabbits were used for *T. pallidum* strain propagation. Animal care was provided in accordance with the Guide for the Care and Use of Laboratory Animals and procedures were conducted under protocols approved by the University of Washington Institutional Animal Care and Use Committee (IACUC) under protocol number 4243-01. Polyclonal antibodies against recombinant TP0435 were produced in BALB/c mice using the previously described protocol[1]. All mouse experiments were performed in accordance with all provisions of the Animal Welfare Act, the Guide for the Care and Use of Laboratory Animals, and the PHS Policy on Humane Care and Use of Laboratory Animals. These experiments were conducted under the protocol number 14011D0617 approved by the Rutgers Biomedical and Health Sciences IACUC. Coded, secondary syphilis patient serum was provided for this work by Dr. Sheila Lukehart.

Plasmid cloning for *B. burgdorferi* transformation. High passage *B. burgdorferi* strains B314 and B31HP[2] were grown in BSKII medium containing 6% rabbit serum at 33°C. A Shuttle vector for *B. burgdorferi* containing a codon-optimized firefly luciferase[3] was used to clone TP0435 of *T. pallidum* with its 500 nucleotides upstream region containing putative promoter using the primers, 5Tp435M-Sac and 3Tp435-Bam with sequences 5'GGCGAGCTCCGTGCTTTCTCG ACTGACGC3' and 5'CGCGGATCCCTATTTCTTTGTTTTTTGAGCACG3', respectively. Restriction enzyme cleavage sites are underlined in the primer sequences. For expression of recombinant TP0435, promoterless *tp0435* gene without the nucleotides encoding the first 25 amino acids of the translational product was amplified using the primers 5Tp435-Nde with sequence GGCCATATGTGCACAACCGTGTGTCCGCA, and 3Tp435-Bam and cloned in pET15b expression vector.

Purification of recombinant Polyhistidine-tagged TP0435. One liter culture of BL21(pLysS) strain of *E. coli* containing *tp0435* gene cloned in pET15b plasmid was grown at 37°C with shaking until absorbance at 600nm reached approximately 0.6, i.e., culture reached mid-logarithmic phase of growth. Expression of polyhistidine-tagged TP0435 protein was induced using 1mM IPTG by incubation at 37°C overnight without shaking. The next day, supernatant was discarded after centrifugation and bacterial pellet resuspended in PBS containing 1mg/ml lysozyme and incubated at 37°C for 30 minutes. Culture was lysed by sonication in the presence of proteinase inhibitor cocktail (Sigma-Aldrich Inc. MO) while placed on ice. The lysed bacterial pellet was removed after centrifugation, supernatant filtered through 0.22µ syringe filter and recombinant TP0435 purified following the manufacturer's kit and protocol (Novagen, WI).

Production of polyclonal antibodies in mice against the recombinant TP0435. Recombinant purified TP0435 was mixed with an equal volume of Complete Freund's Adjuvant (CFA) at 1:1 ratio and emulsion prepared by repeated vortexing in cold. Completion of emulsion preparation was confirmed by 'drop on water' method, such that the emulsion drop remained intact on the surface of water. Each mouse was injected with 0.1ml of emulsion. The step was repeated to provide two booster doses at two weeks interval using Incomplete Freund's adjuvant (IFA) instead of CFA for emulsion preparation. Mice were bled a week after the second booster dose by retro-orbital bleeding and titer of sera determined by Western blotting. After a week rest following the bleeding, mice were given a third and final boost. One week after this injection,

blood was collected by cardiac puncture and mice were euthanized. Sera were separated by centrifugation and stored at -20°C until use.

Immunoaffinity enrichment of TP0435. After washing *B. burgdorferi* B31HP(pTP) culture three times with the suspension buffer (50 mM Tris-HCl, pH7.4, 150 mM NaCl, 5 mM EDTA), lysis was carried out with 1.7% TritonX100 containing suspension buffer i.e., lysis buffer, in the presence of protease inhibitor cocktail (Sigma P8849). After removing lysed bacterial pellet by centrifugation, proteins in the supernatant were precipitated with three volumes of cold acetone and after drying in air, pellet was resuspended in solubilization buffer, i.e., suspension buffer containing 0.2% Triton-X100 and protease inhibitor cocktail. Immunoaffinity enrichment of TP0435 from *B. burgdorferi* was conducted using a two-step process. In the first step, antibodies against *B. burgdorferi* cross-linked with protein G were used to remove majority of Borrelia proteins and supernatant containing significantly reduced level of *B. burgdorferi* proteins recovered after centrifugation. Protein G beads crosslinked to TP0435 mouse antiserum were then used to further enrich TP0435 protein from this supernatant. After washing, the beads boiled in protein loading dye were resolved by 12.5% SDS-PAGE and stained with Sypro Ruby (*T. pallidum*) or silver stain (*B. burgdorferi*). The first enrichment step was eliminated for immunoprecipitation and enrichment of TP0435 from *T. pallidum*. Gel segment containing enriched TP0435 from *B. burgdorferi* (4-5mm) was sent to Tufts University Core facility to determine N-terminal sequence by Edman degradation method.

***T. pallidum* strain propagation and collection for nucleic acid extraction and electron microscopy.** After injection of $5x10^{7}$ *T. pallidum* Nichols strain per testicle, spirochetes were allowed to multiply until rabbit developed an orchitis. At peak orchitis, which is usually detected at 10-12 days post-inoculation, rabbits were euthanized to remove the testicles. Spirochetes were extracted in sterile saline from minced testicular tissue and collected in sterile 15 ml tubes. *T. pallidum* bacteria were immediately centrifuged at 250 xg for 10 minutes to remove rabbit tissue debris. For DNA extraction, treponemes were centrifuged at 12,000 x*g* for 30 min at 4˚C and after removing the supernatant, pellets were resuspended in 200 µl of 1X DNA extraction buffer (10 mM Tris pH 8.0, 0.1 M EDTA, 0.5% sodium dodecyl sulfate). DNA was extracted using the Mini Kit (Qiagen Inc., Chatsworth, CA) according to the manufacturer's instructions, with the exception of adding 50 µl of Proteinase K (from a 100 mg/ml stock solution) and incubating the sample overnight to lyse bacteria. After the final elution step in 200 µl of molecular-grade H_2O, DNA was stored at -20ºC until needed for amplification reactions. For scanning electron microscopy (SEM), equal volumes of treponemal suspension and 2X fixative solutions were mixed. The samples were fixed immediately after recovery from rabbit testes in a buffer containing 2.5% glutaraldehyde, 0.1 M sodium cacodylate buffer pH 7.4, 0.2 M sucrose, and 5 mM $MgCl_2$.

IFA. TP0435 present on the surface or periplasmic region of B31HP(pTP) and B314(pTP) strains was stained using secondary syphilis patient serum (1:100 dilution) obtained from Dr. Sheila Lukehart at University of Washington, or anti-TP0435 mouse antiserum (1:200 dilution) followed by respective secondary antibodies (1:100 dilution) conjugated to Alexa fluor 488 (Life Technologies-Molecular Probes, MA) using the previously described protocol[4]. Staining of DNA with 4',6-diamidino-2-phenylindole (DAPI) was used to detect all bacteria present in any given microscopic field of view. Permeabilization of *B. burgdorferi* with cold methanol for 20 minutes followed by staining determined proteins located in the periplasmic region. Anti-FlaB mouse

monoclonal antibodies (1:50 dilution) followed by of anti-mouse secondary antibodies (1:100 dilution) conjugated with TRITC (Life Technologies-Molecular Probes, MA) were used to stain intact bacteria to ascertain integrity of the outer membrane of spirochetes while staining with anti-FlaB antibodies after permeabilization followed by secondary antibodies were used to detect periplasmic flagellin.

Binding assay using radiolabeled *B. burgdorferi* to mammalian cells. *B. burgdorferi* strains were labeled with a ^{35}S Met-Cys mixture and harvested by centrifugation when the density was between 5×10^7 and 1×10^8 spirochetes/ml. After three five-minute washes with PBS/BSA without shaking to remove unbound label, bacterial pellets were resuspended in 20% glycerol containing BSK-H medium without serum and 1ml aliquots of labeled *B. burgdorferi* were stored at -80°C. Binding assays were conducted using *B. burgdorferi* and mammalian cell lines as previously published[4].

Results

Effect of endogenous plasmid profile of *B. burgdorferi* strains B314 and B31HP. Both B314 and B31HP lack a significant number of endogenous plasmids (Supplementary Fig. S1) rendering them non-infectious such as the loss of plasmid lp25 that encodes for nicotinamidase enzyme, which is essential for survival of Lyme spirochetes in the host. The absence of lp17 and hence, the lack of BBD18 regulator results in over-expression of OspC in B314 strain[5]. Furthermore, absence of lp36 plasmid that possesses fibronectin and glycosaminoglycan binding protein encoding gene *bbk32* in both strains, and particularly the loss of lp54 in B314 results in the absence of adhesins, such as DbpA, DbpB, and severely attenuates ability of B314 strain to bind various mammalian cell lines *in vitro*.

Surface labelling of TP0435 on B31HP(pTP) strain. Secondary syphilis patient serum did not label B31HP(V) control strain without permeabilization indicating that antibodies in the serum did not bind to any protein on the surface of this *B. burgdorferi* strain similar to B314(pTP) strain (Supplementary Fig. S2a). Recognition of TP0435 protein with secondary syphilis patient serum in B31HP(pTP) without permeabilization that becomes more intense on permeabilization of the spirochetes indicates the presence of this protein both on the surface and periplasmic compartment (Supplementary Fig. S2c and S2d, and supplementary Table S1). Integrity of the outer membrane of spirochetes was maintained during IFA since FlaB was labelled with the specific monoclonal antibodies in permeabilized, and not unpermeabilized B31HP(pTP) strain (Supplementary Fig. S2).

Integrity of outer membrane is essential for proper surface labelling of TP0435 in *T. pallidum*. Experimental manipulations such as centrifugation and washing of *T. pallidum* after recovery of these spirochetes from rabbit testes have been previously reported to often compromise outer membrane of these spirochetes[6-8]. We found that integrity of the outer membrane of *T. pallidum* is affected to different extent in different harvests (Supplementary Fig. S4 and Fig. S5d). Compromised outer membrane results in more pronounced labelling of TP0435 in different *T. pallidum* cells by Immuno-SEM (Supplementary Fig. S4).

Predicted model for production of TP0435 isoforms. It is to be noted that several *T. pallidum* predicted and known lipoproteins, unlike *B. burgdorferi* lipoproteins, possess multiple cysteines in the first 50 amino acids of the predicted translation products, suggesting possibility differential post-translational modification (Supplementary Table S2). Lipoprotein computational prediction

suggests that three residues, Cys22, Cys25, and Cys29 in TP0435 are possibly the fourth amino acid of three potential lipoboxes (SAL**C**, CVS**C**, and TTV**C**), which can be targeted by the type II signal peptidase in spirochetes[9] and result in lipidated isoforms production (Supplementary Fig. S5a). Molecular weights of these three isoforms with Cys22, Cys25, and Cys29 as the first residues without lipidation were calculated to be 14.5, 14.2, and 13.8 kD, respectively. Different TP0435 isoforms seen in *B. burgdorferi* are unlikely to be degradation products of TP0435 since these protein bands are larger than the predicted sizes and the prominent mature exported protein band detected in *T. pallidum.* Brautigam and co-workers crystallized a recombinant TP0435 protein that lacks the first 32 amino acids from their structure[10]. The missing segment in their structure contains three cysteines, Cys-22, Cys-25, and Cys-29. Therefore, we have used the predicted TP0435 using Phyre 2 Program instead that almost completely resembles TP0435 structure determined by Brautigam et al.[10] but possesses the N-terminal region (Supplementary Fig. S5a and S5b).

Lack of opsonophagocytosis of B314(V) and B31HP(V) control strain using anti-TP0435 antibodies. Mouse macrophage cell line J774A.1 fails to phagocytise B314(V) and B31HP(V) even after 6h when these strains were opsonized with anti-TP0435 mouse antibodies, as expected, because these antibodies do not recognize surface proteins of these control strains.

References

1 Parveen, N. & Leong, J. M. Identification of a candidate glycosaminoglycan-binding adhesin of the Lyme disease spirochete *Borrelia burgdorferi*. *Molecular Microbiology* **35**, 1220-1234 (2000).

2 Sadziene, A., Wilske, B., Ferdows, M. & Barbour, A. The cryptic *ospC* gene of *Borrelia burgdorferi* B31 is located on a circular plasmid. *Infect Immun* **61**, 2192-2195 (1993).

3 Blevins, J. S., Revel, A. T., Smith, A. H., Bachlani, G. N. & Norgard, M. V. Adaptation of a luciferase gene reporter and lac expression system to *Borrelia burgdorferi*. *Appl Environ Microbiol* **73**, 1501-1513 (2007).

4 Fischer, J. R., Parveen, N., Magoun, L. & Leong, J. M. Decorin-binding proteins A and B confer distinct mammalian cell type-specific attachment by Borrelia burgdorferi, the Lyme disease spirochete. *Proc Natl Acad Sci U S A* **100**, 7307-7312 (2003).

5 Sarkar, A., Hayes, B. M., Dulebohn, D. P. & Rosa, P. A. Regulation of the virulence determinant OspC by bbd18 on linear plasmid lp17 of *Borrelia burgdorferi*. *Journal of bacteriology* **193**, 5365-5373, doi:10.1128/JB.01496-10 (2011).

6 Cox, D. L., Chang, P., McDowall, A. W. & Radolf, J. D. The outer membrane, not a coat of host proteins, limits antigenicity of virulent *Treponema pallidum*. *Infection and Immunity* **60**, 1076-1083 (1992).

7 Stamm, L. V., Hodinka, R. L., Wyrick, P. B. & Bassford, P. J., Jr. Changes in the cell surface properties of Treponema pallidum that occur during in vitro incubation of freshly extracted organisms. *Infect Immun* **55**, 2255-2261 (1987).

8 Radolf, J. D., Fehniger, T. E., Silverblatt, F. J., Miller, J. N. & Lovett, M. A. The surface of virulent Treponema pallidum: resistance to antibody binding in the absence of complement and surface association of recombinant antigen 4D. *Infect Immun* **52**, 579-585 (1986).

9 Setubal, J. C., Reis, M., Matsunaga, J. & Haake, D. A. Lipoprotein computational prediction in spirochaetal genomes. *Microbiology* **152**, 113-121 (2006).
10 Brautigam, C. A., Deka, R. K., Liu, W. Z. & Norgard, M. V. Insights into the potential function and membrane organization of the TP0435 (Tp17) lipoprotein from *Treponema pallidum* derived from structural and biophysical analyses. *Protein science : a publication of the Protein Society* **24**, 11-19 (2015).

Supplementary Table S1. Total fluorescence intensity in the microscopic field of view in the Fig. 1 and in the supplementary Fig. S2.

Sample No.	Fluorescence Intensity Min.	Fluorescence Intensity Max.	Average Fluorescence intensity	Average SS2/DAPI
B314(V)–SS Unperm.	193	511	**249**	0.59
B314(V)–DAPI Unperm	281	4095	424	
B314p(V)–SS Perm.	179	1524	**243**	0.59
B314p(V)–DAPI Perm.	316	4095	415	
B314(pTP)–SS Unperm.	225	4095	**381**	0.73
B314(pTP)–DAPI Unperm.	365	4095	520	
B314(pTP)–SS Perm.	412	4095	**936**	1.70
B314(pTP)–DAPI Perm.	280	4095	549	
B31HP(V)–SS Unperm.	207	1819	**265**	0.70
B31HP(V)–DAPI Unperm.	305	4095	380	
B31HP(V)–SS Perm.	177	1606	**234**	0.31
B31HPp(V)–DAPI Perm.	595	4095	757	
B31HP(pTP)–SS Unperm.	233	4095	**348**	0.93
B31HP(pTP)–DAPI Unperm.	255	4095	373	
B31HP(pTP)–SS Perm.	317	4095	**553**	0.98
B31HP(pTP)–DAPI Perm.	317	4095	562	

Supplementary Table S2. ***T. pallidum*** **surface protein sequences with multiple cysteines in the first fifty amino acids**

S. No.	Lipoprotein	First fifty amino acid sequence	# of Cysteines.
1.	TP0034*	MQRCSVVAALAGVVFLAQACSLSTPSRITHTDKLPVVVTFNALKELTQMV	2
2.	TP0074*	MCFARSRLFSRGAVGTVCCTVLFLACRVRTPSSVPLRSGSVRAAVPEATS	4
3.	TP0104*	MKRFIPHRVIHAVCIGLALVGCRKLDSRAGDFELTIIHINDHHSHLEPEP	2
4.	TP0113*	MRIPKWTPATWSVVAGCIGGVLGIVIVGIASPIRIISPTDNGVVTRFGKY	1
5.	TP0133*	MMARSRCVHRVVHQAACIGVIGLSTSALTTCDFTGIFVAIQSEVPIKTPS	3
6.	TP0136*	MGRSTMDTQYMRRRVCTVVRAVVCLLSTSLLTTCDFTGIFAAIQSEVPIK	3
7.	TP0144*	MAHRMRVVFCCAAALSLCCCSRSRTDAPSLVIYVYDSFASEWGPGPELAR	5
8.	TP0171 (Tpp15)	MVKRGGAFALCLAVLLGACSFSSIPNGTYRATYQDFDENGWKDFLEVTFD	2
9.	TP0226*	MFFSLYERRQSLLHRAPPVAKVASFALLLLCCNAQSWCMHASLTLVLLAL	3
10.	TP0248*	MWHLRCSNWRGSGVFGMCFSLSGCVMGQWCVSRAIVRGHTQGGAVREVVR	4
11.	TP0257*	MRGTYCVTLWGGVFAALVAGCASERMIVAYRGAAGYVPEHTFASKVLAFA	2
12.	TP0260*	MCYYRYVQVHSIWRRFCALGLLVPFLLLLFSCTNTVGYGVLQWSLPDLGL	3
13.	TP0319 (TmpC)*	MREKWVRAFAGVFCAMLLIGCSKSDRPQMGNAGGAEGGDFVVGMVTDSGD	2
14.	TP0405 McbG*	MYAPRMRLNRVDFSGFHFVGCNFFNTVFNACVFANVVMRMCFFERCYLVN	4
15.	TP0410*	MSKKARFGVVLVVLAACSGFLFPTLQWYFLTDAQTRQRALSSREQIKEYA	1
16.	TP0435 (Tpp17)	MKGSVRALCAFLGVGALGSALCVSCTTVCPHAGKAKAEKVECALKGGIFR	4
17.	TP0453*	MIRRRYRGCTQGAWIVSVGMLFASCTSGAWKASVDPLGVVGSGADVYLYF	2
18.	TP0455*	MPTLRWQGWRMKLKRSLIVGGGLLLCCAHGYAQAKGARASVHIAYHNRTI	2
19.	TP0456*	MNTRLALVLCAVGSGVLSFSCARTAEPTPAASTHVPVTTAGALSVTPPSS	2
20.	TP0462*	MRRIVCPPVLFLSASLLTGCDFSGIFASIQSEVPLKIPSIRGVVTGLVKC	3
21.	TP0486* P83/P100	MKNVLPMCAVLGAGCLFALEVDRRELERANATVEFENFAGTHTDVDSAAA	2
22.	TP0572*	MSALFSLVAVYVLVCALHKQIKKYASVCYLGSACVSVAVVCVVWSGATKG	4
23.	TP0574*	MKVKYALLSAGALQLLVVGCGSSHHETHYGYATLSYADYWAGELGQSRDV	1
24.	TP0622*	MGALTKSIDRAIGVSVGFVLIPVFLCCRSSSKVVPTRAGTDPVSSASQAP	2
25.	TP0646*	MGRMRKVACAFRKLWCRFVVLSISILVSCSVVTRHPLRLGFFTFFLLGSF	3
26.	TP0655 PotD*	MKRFCVSSSRILSLLFLSLWMGSCLQTRQDVLYLYNWTYYTPTSLIKKFE	2

27.	TP0684*	MKENSCTACSRRLALFVGAAVLVVGCSSKTDVTLNRDKPLVFFNRQPSDP	3
28.	TP0693	MDRFFCTVWVWSVLFGACTSQTRSSFSLNADGLNSSGVAHASEHVSHAAA	2
29.	TP0773*	MRNKVRVLAVVAALAAACAVGFFLGRWFDFSARSSVLEAADSLSVSSSEA	1
30.	TP0784*	MRIRLLFAPVSCLLLVCCADSSALRWIQQPSATRNTPTIAFFQVTFDRYN	3
31.	TP0796*	MKSSCVYWRIGVLVCILCGVGSCGGRARVREYSRAELVIGTLCRVRVYSK	3
32.	TP0821 Tpn32*	MKGKTVSAALVGKLIALSVGVVACTQVKDETVGVGVLSEPHARLLEIAKE	1
33.	TP0839*	MDKKARAHTVIVCLVGALSLACAYLLGGSMKYLRGDVSRFWTSSRMQRHP	2
34.	TP0862* FklB	MILKKEAAGVCSFALLLLAGMSIFSCRKGAEGVSSAGEAADKKALTADQL	2
35.	TP0895*	MCRRLCHHRWRVWGKVASLGVLLLGGLVACTSSAAGSTSNTRPGVRMTIT	2
36.	TP0938*	MKFCRGWRGARAGQFARLLCVCWCSLGACSCVRESTERSGVVPISPSSQQ	6
37.	TP0954	MRYGTLFKVSVLLGALFVSCVSTGSNSARESERAQLLKSENPNIRFAAQL	1
38.	TP0956*	MKHPSVRVCCFAFASCLLCAGCSLKRLAFSSLSHTLAPFPEGELDAHLSD	4
39.	TP0971 Tpd*	MKRVSLLGSAAIFALVFSACGGGGEHQHGEEMMAAVPAPDAEGAAGFDEF	1
40.	TP1041 ClpPII*	MLFKRSFYATLSGSCLVWGCCATVYREHAREKWGVGVVMRCDATQEKRAH	4

* Indicate predicted lipoprotein based upon SpLip program. According to SpLip position-specific scoring matrix, all bacterial lipoproteins contain amino-terminal cysteine (+1 position) with lipid modification. Spirochete lipobox consists of five amino acids at the carboxy terminal end of the signal peptide cleaved by signal peptidase II. Its characteristics are that; (1) at -1 position (carboxy-terminal amino acid of signal peptide), Ala, Gly, Ser, Asn or Cys are allowed. (2) At -3 and/or -4 position, Leu, Ile, Val and Phe should be present. Charged amino acids, Lys, Arg, His, Asp, and Glu are forbidden in the lipobox.

Several lipoproteins are predicted to possess more than one cysteine residue, follow the rules 2 and 3 but may possess Val at -1 position, suggesting that the alternate cleavage may also occur in these lipoproteins similar to TP0435 by signal peptidase II.

Supplementary Fig. 1

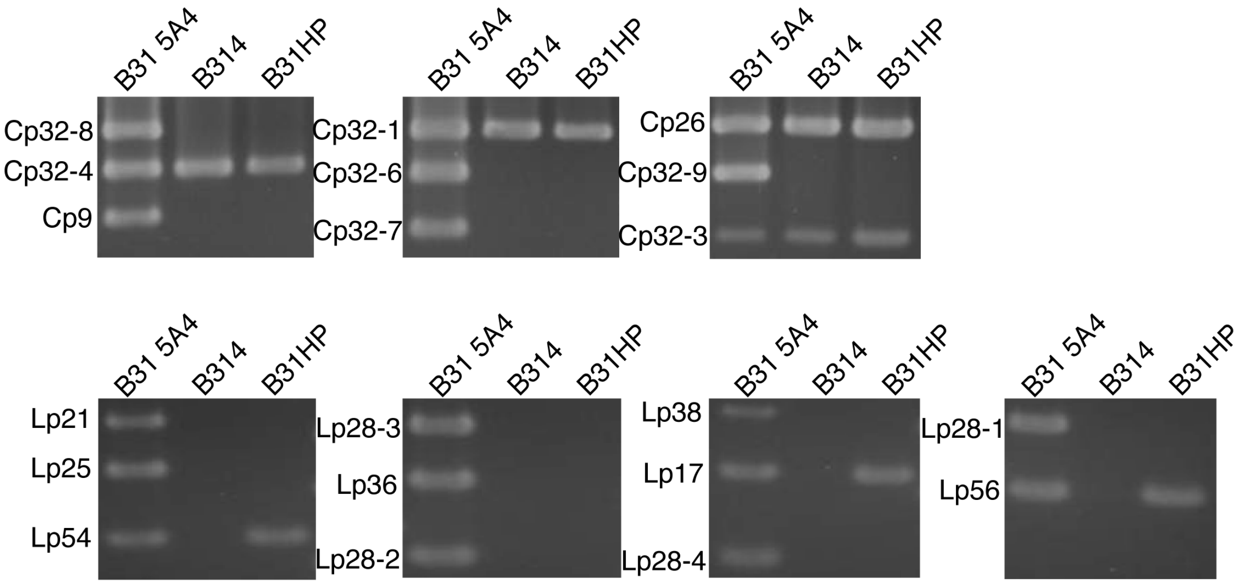

Fig. S1. Comparison of endogenous circular (Cp) and linear (Lp) plasmid profile of B314 and B31HP strains with the infectious B31 5A4 strain of *B. burgdorferi*. B314 retains only four endogenous plasmids Cp26, Cp32-1, Cp32-3, Cp32-4 and lacks all linear plasmids, while B31HP contains Cp26, Cp32-1, Cp32-3, Cp32-4, Lp17, Lp54, and Lp56. Although the *ospC* gene is located on Cp26 plasmid, genes encoding regulators of OspC expression are located in trans on Lp17 plasmid and prevent over expression of OspC. Significant numbers of genes located on Lp54 plasmid have been reported to express surface lipoproteins of *B. burgdorferi* including the major adhesins, OspA, OspB, DbpA and DbpB.

Supplementary Fig. S2

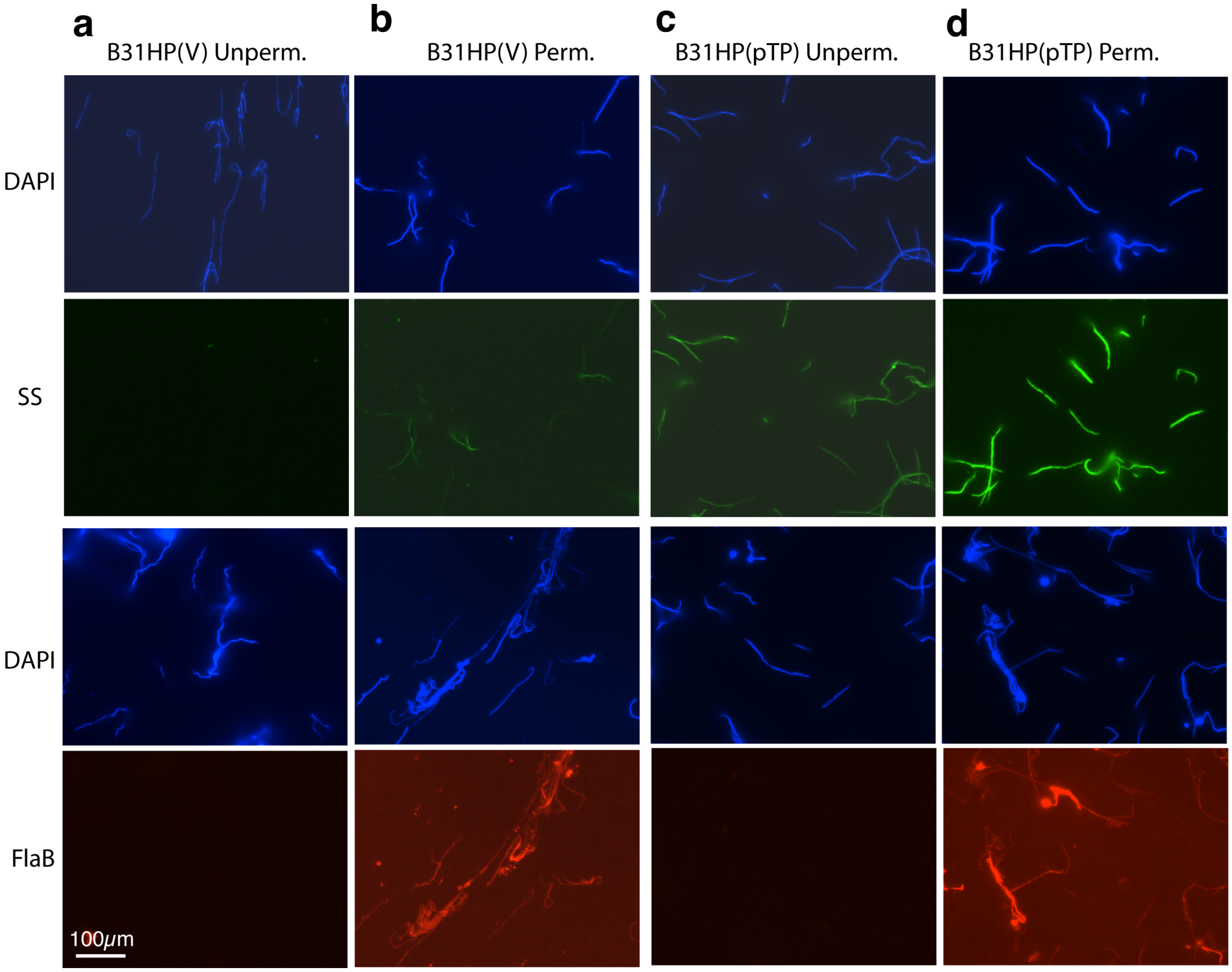

Fig. S2. TP0435 is also expressed on *B. burgdorferi* strain B31HP surface. (a) Absence of staining of surface proteins in intact B31HP(V) control strain by secondary syphilis (SS) in IFA indicates the lack of cross-reactivity of antibodies with even this *B. burgdorferi* strain surface proteins. All spirochetes in the respective fields were imaged after simultaneous staining with DAPI. Lack of periplasmic flagellar staining with anti-FlaB antibodies followed by anti-mouse TRITC conjugate reveal that the integrity of outer membrane of spirochetes is maintained during IFA. Permeabilization also allows prominent staining of flagellin. **(b)** Poor staining of B31HP(V) with SS patient serum on permeabilization indicates its weak reactivity with some periplasmic proteins of *B. burgdorferi*. **(c)** Punctate staining of majority of *B. burgdorferi* strain B314(pTP) with SS serum in IFA, and not of flagellar protein with anti-FlaB antibodies indicates the presence of TP0435 on the spirochete surface. **(d)** Permeabilization of B314(pTP) results in more intense staining of TP0435 with SS, indicating significant presence of this protein also in periplasmic region. Prominent staining of flagella with anti-FlaB antibodies is also observed on permeabilization. Scale represents all panels in the figure.

Supplementary Fig. S3

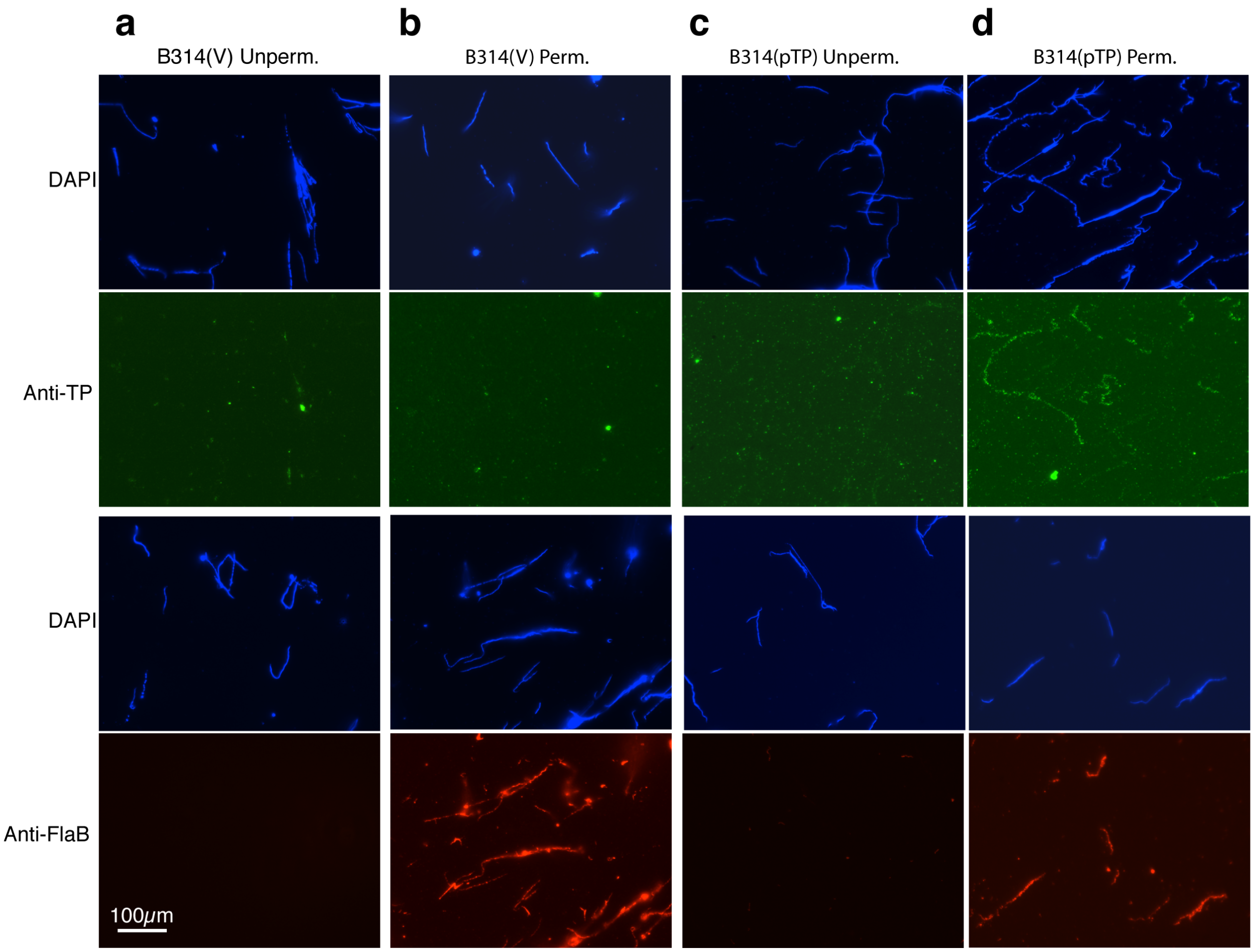

Fig. S3. TP0435 could not be detected on the surface of *B. burgdorferi* by IFA using antibodies raised against recombinant TP0435. (a and b) B314(V) control showed no reaction with anti-TP0435 antibodies raised against recombinant protein with or without permeabilization while flagella staining was detected only after permeabilization, as expected. **(c and d)** High background fluorescence made it difficult to discern surface labelling with anti-TP0435 antibodies on B314(pTP) while punctate staining was detected on permeabilization of this strain. Lack of flagellar staining without permeabilization indicates that integrity of spirochetes was maintained during IFA. Scale represents all panels in the figure.

Supplementary Fig. S4

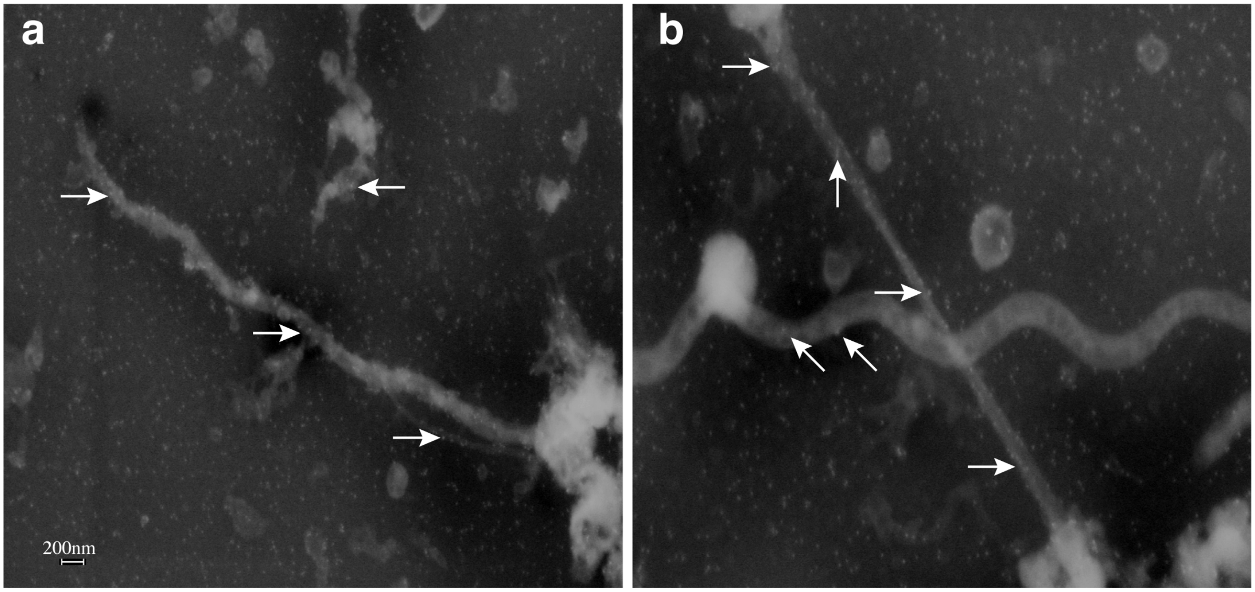

Fig. S4. Proper surface staining of TP0435 on *T. pallidum* requires that integrity of outer membrane remains intact. (a and b) Gold particles attached to *T. pallidum* in a more pronounced manner, as indicated by arrows, when outer membrane is uneven and apparently tattered suggesting that mouse anti-TP0435 antibodies recognized periplasmic TP0435 more efficiently in all spirochetes in the field. *T. pallidum* with apparent intact outer membrane show only few gold particles. Higher gold particles observed in the background are likely due to the labelling of fragmented outer membrane present in the field. Scale represents both panels in the figure.

Supplementary Fig. S5

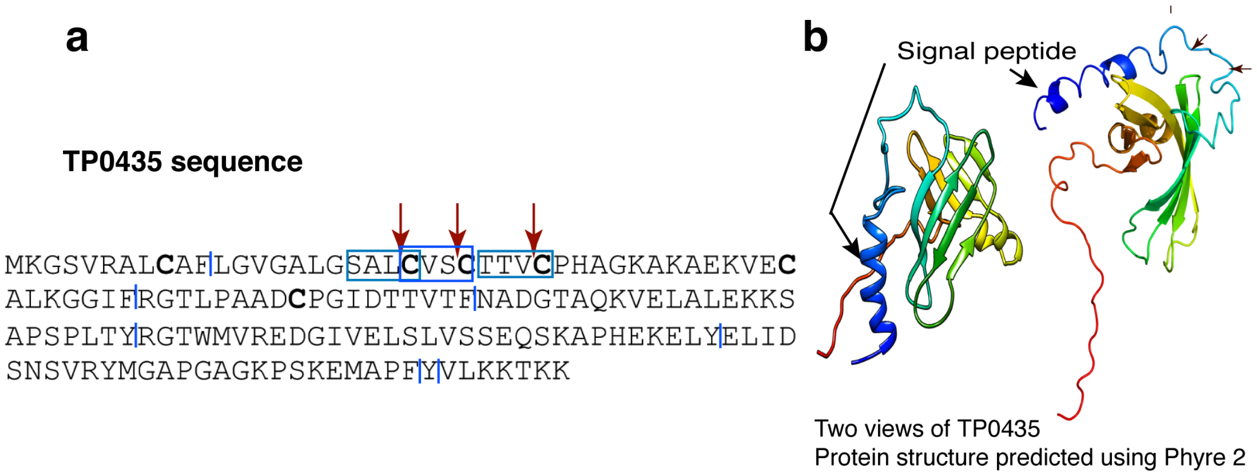

Fig. S5. Sequence analysis and structure of TP0435 predicting differential processing. (a) Open Reading Frame of TP0435 derived from *T. pallidum* genome sequence with vertical blue lines marking the chymotrypsin cleavage sites and red arrows depicting the cysteine residues in predicted lipoboxes shown by blue boxes. **(b)** Two views of the structure of TP0435 predicted by the web Phyre 2 analysis show eight β-sheets, similar to those observed in outer membrane lipoproteins of other spirochetes with red arrows marking the cysteines in the lipoboxes.

Supplementary Fig. S6

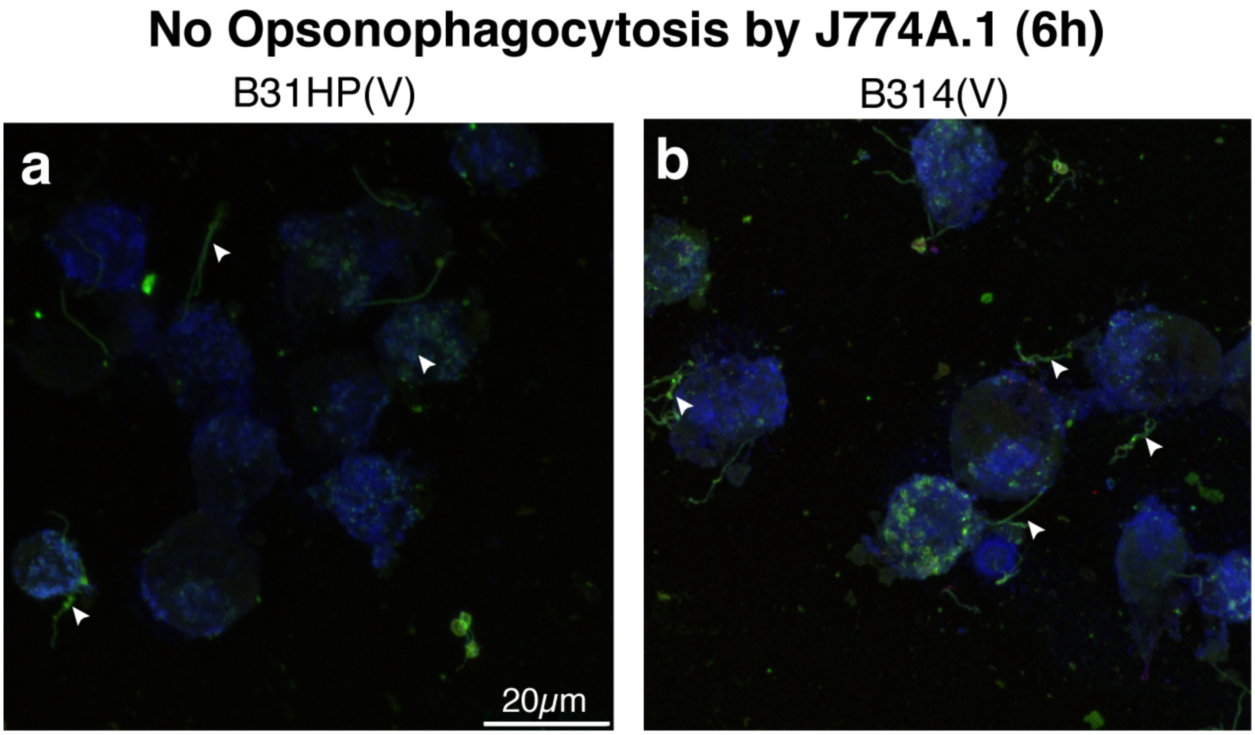

Fig. S6. Anti-TP0435 antibodies do not allow opsonophagocytosis of B314(V) and B31HP(V) control strains. (a and b) No phagocytosis of B31HP(V) and B314(V) control strains preincubated with anti-TP0435 mouse antibodies was detected even after 6h co-incubation of opsonised spirochetes with J774A.1 mouse macrophage cell line. Extracellular spirochetes (arrowhead, green/yellow) bound to macrophages are observed on staining of *B. burgdorferi* before permeabilization, while no intracellular spirochetes (red) are detected on counterstaining after permeabilization with antibodies against *B. burgdorferi* crude OMV preparation followed by TRITC conjugated secondary antibodies. Scale represents both panels in the figure.
